# Endotracheal tubes and fluid aspiration: an in vitro evaluation of new cuff technologies

**DOI:** 10.1186/s12871-017-0328-0

**Published:** 2017-03-04

**Authors:** Maryanne Z. Mariyaselvam, Lucy L. Marsh, Sarah Bamford, Ann Smith, Matt P. Wise, David W. Williams

**Affiliations:** 1 Oral and Biomedical Sciences, School of Dentistry, Cardiff University, Heath Park, Cardiff, Wales CF14 4XY UK; 2 School of Biosciences, Cardiff University, Cardiff, Wales UK; 3 Adult Critical Care, University Hospital of Wales, Cardiff, Wales UK

**Keywords:** Ventilator-associated pneumonia, Nosocomial pneumonia, Endotracheal tube cuff, Endotracheal tube, Pulmonary aspiration

## Abstract

**Background:**

Aspiration of subglottic secretions past the endotracheal tube (ETT) cuff is a prerequisite for developing ventilator-associated pneumonia (VAP). Subglottic secretion drainage (SSD) ETTs reduce aspiration of subglottic secretions and have demonstrated lower VAP rates. We compared the performance of seven SSD ETTs against a non-SSD ETT in preventing aspiration below inflated cuffs.

**Methods:**

ETTs were positioned vertically in 2 cm diameter cylinders. Four ml of a standard microbial suspension was added above inflated cuffs. After 1 h, aspiration was measured and ETTs demonstrating no leakage were subjected to rotational movement and evaluation over 24 h. Collected aspirated fluid was used to inoculate agar media and incubated aerobically at 37 °C for 24 h. The aspiration rate, volume and number of microorganisms that leaked past the cuff was measured. Experiments were repeated (×10) for each type of ETT, with new ETTs used for each repeat. Best performing ETTs were then tested in five different cylinder diameters (1.6, 1.8, 2.0, 2.2 and 2.4 cm). Experiments were repeated as above using sterile water. Volume and time taken for aspiration past the cuff was measured. Experiments were repeated (×10) for each type of ETT. Results were analysed using non-parametric tests for repeated measures.

**Results:**

The PneuX ETT prevented aspiration past the cuff in all experiments. All other ETTs allowed aspiration, with considerable variability in performance. The PneuX ETT was statistically superior in reducing aspiration compared to the SealGuard (*p* < 0.009), KimVent (*p* < 0.002), TaperGuard (*p* < 0.004), Lanz (*p* < 0.001), ISIS (*p* < 0.001), SACETT (*p* < 0.001) and Soft Seal (*p* < 0.001) ETTs. Of the 4 ETTs tested in differing cylinder sizes, the PneuX significantly reduced aspiration across the range of diameters compared to the SealGuard (*p* < 0.0001), TaperGuard (*p* < 0.0001) and KimVent (*p* < 0.0001) ETTs.

**Conclusions:**

ETTs showed substantial variation in fluid aspiration, relating to cuff material and design. Variability in performance was likely due to the random manner in which involutional folds form in the inflated ETT cuff. The PneuX ETT was the only ETT able to consistently prevent aspiration past the cuff in all experiments.

## Background

In the Intensive Care Unit (ICU), ventilator associated pneumonia (VAP) is the commonest infective nosocomial cause of mortality. In addition to the reports of mortality directly attributable to VAP, VAP increases the duration of mechanical ventilation, length of stay and cost [1]. A prerequisite for developing VAP is aspiration of subglottic secretions past the endotracheal tube (ETT) cuff, the extent of which is dependent on the ETT design [2, 3].

The inflated ETT cuff seals the airway, allowing ventilation to only occur though the tube lumen, and prevents movement of air and fluid around the ETT [4]. However, the ETT subverts the patient’s normal pulmonary defence mechanisms, including mucociliary clearance and the cough reflex [2, 5]. After intubation, the ETT, oropharyngeal surfaces and secretions rapidly become colonised with pathogenic bacteria [2, 6]. Gastric contents reflux into the oropharynx, mix with these secretions [7] and accumulate above the cuff. If the airway seal is compromised, aspiration of these secretions occurs [4]. High bacterial load, with chemical and enzymatic injury from gastric secretions, [3] can overwhelm pulmonary defences leading to microbial colonisation of the lower respiratory tract and VAP. One study showed that the bacteria in subglottic secretions were identical to the causative agents of VAP in 70% of patients [8]. Therefore, correctly achieving and maintaining the airway seal is critical in preventing VAP.

**Fig. 1:**
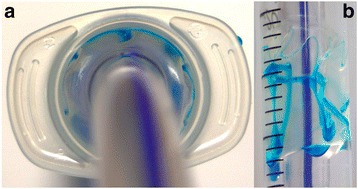
A high-volume-low-pressure cuff inflated to 30 cmH_2_0 inside a 2.0 cm ‘trachea’. The blue dye is placed above the inflated cuff to demonstrate aspiration of fluid past the cuff. Despite inflating the ETT cuff to the correct tracheal wall pressure, it is only partially inflated with excess material folding and causing involutions. The blue dye highlights the formation of channels, which allow leakage of subglottic fluid into the lungs overhead view (**a**) and a lateral view (**b**)

Conventionally, ETTs have high-volume, low-pressure (HVLP) cuffs [9]. The fully inflated HVLP ETT cuff diameter is larger than the adult trachea [10], and this design prevents tracheal mucosal injury by allowing the pressure within the cuff to be equal to the tracheal wall pressure. Therefore, the cuff is only partially inflated, even when correct pressures are used. The excess material folds and causes involutions, causing channels to develop (Fig. 1) [9]. These channels facilitate aspiration of subglottic secretions into the lungs. It is widely accepted that aspiration occurs for all HVLP ETTs to varying degrees [11] and this has been consistently demonstrated in in vitro and clinical studies [10, 12–17].

Manufacturers have redesigned their ETTs and cuffs in order to prevent aspiration of subglottic secretions. For example, ETTs have been developed that allow removal of subglottic fluids, thereby reducing the available volume of secretions entering the lungs. These subglottic secretion drainage (SSD) ETTs, have demonstrated lower VAP rates [12, 18–24]. Other ETTs use novel cuff materials to reduce the number or size of channels [25], variations to the cuff shape, employ devices to continuously maintain cuff pressure [26] or employ antibacterial coatings [27]. However, in ETT design, manufacturers must account for the variability in the size and shape of human tracheas. Tracheas are tapered and exhibit dimensional variability. The anterior-posterior (AP) tracheal diameter ranges between 1.27-2.38 cm for women and 1.68-2.86 cm for men [28]. Selecting the correct size ETT for patients is subjective and determined on the patient’s sex, height or weight [29]. The wrong size can cause tracheal ischemia (if too large) or facilitate aspiration (if too small) [29]. A recent study identified an inverse relationship between tracheal size and body mass index [30], highlighting potential difficulties in making accurate assessments. Therefore, ETTs need to account for anatomical variation and safeguard the trachea.

This in vitro study aimed to compare ‘new generation’ ETTs in the prevention of aspiration of fluids past the cuff in ‘tracheal’ models. Experiments used microbial suspensions to highlight passage of microorganisms past the cuff, and a physiological range of model ‘tracheas’ to determine whether size affected the degree of microaspiration.

## Methods

### Aspiration of microbiological fluids

#### Preparation of microorganisms

Test microorganisms were *Pseudomonas aeruginosa* ATCC 15692, *Staphylococcus aureus* NCIB 9518 and *Candida albicans* ATCC 90027. Bacteria and *Candida* were cultured on blood or Sabouraud’s dextrose agar, respectively. Microorganisms were subcultured and grown overnight (18 h) in Tryptic Soy Broth at 37 °C. Cells were harvested by centrifugation, and the pellets washed in phosphate buffered saline (PBS). This was repeated (×2) and microorganisms were re-suspended in PBS to a turbidity of 0.1 OD. One ml of each bacterial and candida suspension was combined and added to 1 ml of PBS to produce a 4 ml inoculum. On each experimental day, a starting concentration equating to 0.1 OD was used. To minimise variation in the number of viable cells between experiments on different occasions, the same number of colonies were inoculated into Tryptic Soy Broth and incubated for the same period of time (18 h) before preparation and adjustment in PBS.

#### ETT model

**Fig. 2:**
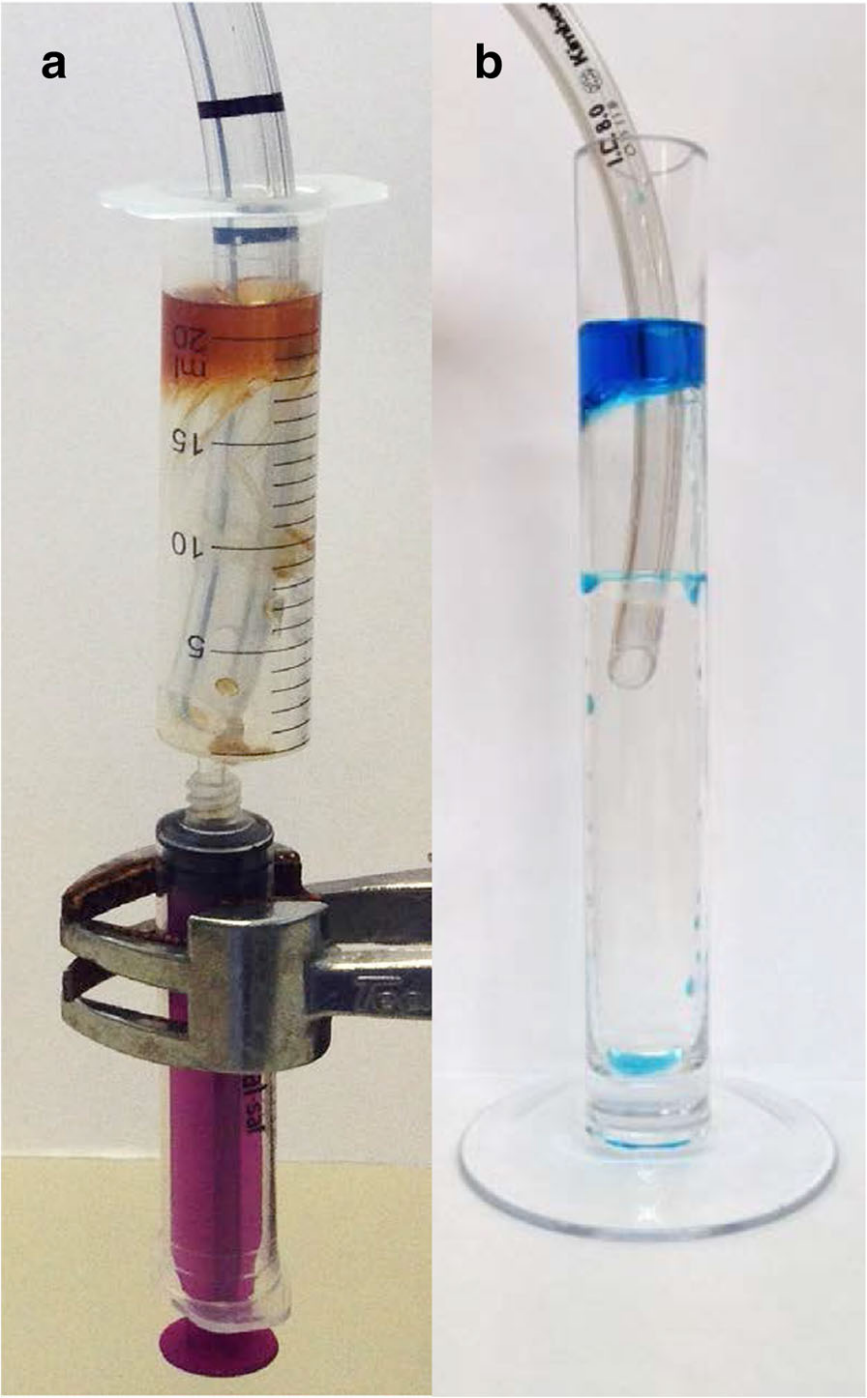
**a** The in vitro trachea model used in the aspiration of microbial fluid study. The 20 ml syringe was connected to the 10 ml enteral syringe via the tip. The endotracheal tube was placed in the 20 ml syringe. Fluid leaking past the cuff was collected aseptically in the enteral syringe. **b** the in vitro trachea model used in the range of tracheas study. The endotracheal tube was placed inside the rigid glass ‘trachea’ and the fluid leaked past the cuff was collected inside the model

A ‘model trachea’ was developed (Fig. 2a) by aseptically connecting the tip of a 20 ml syringe barrel (Becton Dickinson Plastipak, County Louth, Ireland) to a 10 ml enteral syringe (barrel and plunger) (Enteral UK, Yorkshire, UK). The 20 ml syringe barrel, representing the trachea had a 2 cm internal diameter into which the ETT was placed and the enteral syringe was used to capture the aspirated secretions. Seven ETTs (8.0 mm) with design variations to prevent leakage of subglottic secretions and a standard non-SSD ETT (8.0 mm) were selected for testing (Table 1).

**Table 1:** Properties of the ETTs used in the study and the novel technologies used to prevent aspiration of subglottic secretions

ETT and cuff pressure monitor Manufacter	Portex, Smiths Medical, Kent, UK		Venner, Jo Koon Circle, Singapore	Mallinckrodt, Covidien Massachusetts, USA			Kimberly-Clark, Georgia, USA	Teleflex UK, Athione, Ireland
ETT	Portex Soft Seal ® Cuff Tracheal Tube	Portex SACETT™ Suction Above ET Cuff	Venner™ PneuX P.Y.™ ETT	Mallinckrodt™ SealGuard™ Evac Endotracheal Tube	Mallinckrodt™ TaperGuard™ Evac Oral Endotracheal Tube	Mallinckrodt™ Hi-Lo Oral Endotracheal Tube, Lanz System	KimVent™ MICROCUFF™ Subglottic Suctioning Endotracheal Tube	Teleflex ISIS® HVT™
Cuff type	HVLP	HVLP	LVLP	HVLP	HVLP	HVLP	HVLP	HVLP
Cuff material	PVC	PVC	Silicone	Poly-urethane	PVC	PVC	Poly-urethane	PVC
Cuff shape	Cylindrical	Tapered	Cylindrical	Tapered	Tapered	Cylindrical	Cylindrical elongated	Tapered
Cuff resting diameter (cm)	3	3	Dependent on tracheal size	2.7	2.54	3.3	3	2.8
Recommended Cuff Pressure (cm H_2_0)	30	30	80 (=30)	20-30	20-30	25-33	20-30	20-30
Subglottic port(s)	0	1	3	1	1	0	1	1, detachable
Cuff pressure monitor	No	Pressure Easy	Tracheal Seal Monitor	No	No	Lanz valve inflated to 30-34 cm H2O	No	No

#### Assessment of aspiration

Experiments were conducted at 37 °C in a temperature and humidity controlled environment. The distal cuffed ETT was aseptically placed inside the 20 ml syringe barrel and the cuff inflated to the correct pressure using a hand-held manometer (Portex, Smiths Medical International Ltd, Kent, UK) according to the manufacturer’s instructions. If continuous cuff pressure monitors were recommended, these were used to maintain cuff inflation during experiments.

Four ml of standardised microbial suspension was added above the ETT cuff. The time taken for the fluid to leak past the ETT cuff was measured. Any fluid that leaked past the cuff within 1 h, was collected in the enteral syringe and this was recorded as a tube leak. In cases of no observed fluid leak, a standard movement test (140° axial rotation, to mimic oral care movements) was applied to the proximal end of the ETT after the initial 1 h period at 0, 15, 30 and 45 min. If no leak was evident after the movement tests, the enteral syringe was disconnected from the tip of the 20 ml syringe barrel, aseptically filled with 4 ml of sterile PBS and re-connected to the tip of the 20 ml syringe barrel. The 4 ml sterile PBS was then injected into the 20 ml syringe below the inflated ETT cuff. The PBS was withdrawn and the fluid processed to determine potential ‘micro leaks’.

The collected microbial fluid was serially decimal diluted in PBS. Fifty μl of the diluted suspensions were plated on to Tryptone Soya Agar and incubated aerobically at 37 °C for 24 h. The resulting number of colony forming units (cfu) were then counted. Experiments were repeated (×10) for each ETT type and new ETTs were used for each repeat.

ETTs that did not leak during any of the static or movement tests were subjected to a 24 h static test. Experiments were repeated (×3), with new ETTs used for each repeat.

### Effect of ‘tracheal size’ on aspiration

The 4 ‘best performing’ ETTs from the microbiological study were analysed in these experiments. Model tracheas were developed using rigid glass cylinders, with internal diameters of 1.6, 1.8, 2.2 and 2.4 cm (Dabble Labs UK, Kent UK) (Fig. 2b). For the 2.0 cm sized ‘trachea’ diameter, the syringe model described above (and shown in Fig. 2a) was utilised. ETTs were positioned vertically and the study was conducted as outlined above. However, in these studies, 4 ml of sterile water (B. Braun Melsungen AG, Melsungen, Germany) was added above the ETT cuff. The volume and time taken for leakage past the cuff was measured. Experiments were conducted over a 1 h period. If no aspiration was observed, a standard movement test was applied to the proximal ETT as previously described. Experiments were repeated (×10) for each tracheal size.

### Statistic analysis

Statistical analysis was performed using the R Project Model for statistical analysis (The R Foundation, 2014).

#### Aspiration of microbiological fluids

Results were analysed using ANOVA analysis and a Tukey multiple comparison of the means. This test compared the overall performance of all ETTs in preventing aspiration of microbial fluids past the cuff and compared the volume (ml/min) and quantity of cfu that leaked past the cuff over time (cfu/min). Further analysis was performed using non-parametric statistical tests for repeated measures using a pairwise Mann–Whitney *U* Test.

#### Effect of ‘tracheal size’ on aspiration

Results were analysed using non-parametric statistical analysis for repeated measures and stratifying by size of the glass trachea using the Wilcoxon-Nemenyi-McDonald-Thompson test [31], comparing the ETTs and the fluid volume leaked past the cuff (ml/min).

## Results

### Aspiration of microbiological fluids

**Fig. 3:**
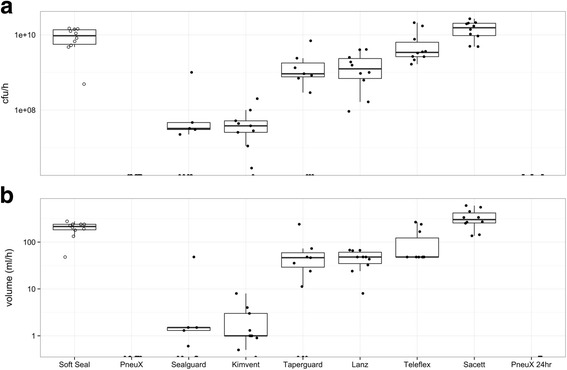
Aspiration of microbial fluid study. ETTs used in the study are shown on the x-axis and include the results of the 24 h study. **a** Log of colony forming units aspirated past the cuff for each ETT. **b** Volume of fluid leaked past the ETT on a logarithmic scale. Results demonstrate that the PneuX ETT was the only tube that prevented aspiration of bacteria in the static, movement and 24 h study. Variability of the results occurred between repeats of the HVLP ETTs

Aspiration was expressed as total cfu/min and volume of fluid leaked past the cuff. With the exception of the PneuX ETT, all ETTs demonstrated leakage of microorganisms past the cuff. The Portex Soft Seal, Lanz, ISIS and SACETT ETTs leaked 4 ml of microbial fluid on all occasions (between <5 min and 1 h). The SealGuard, KimVent and TaperGuard ETTs leaked on some occasions and these were analysed in the movement test. Once rotation was applied, aspiration of bacterial fluid occurred with all TaperGuard and KimVent ETTs and on some occasions with the SealGuard ETT. The PneuX ETT was the only ETT that progressed to the 24 h test. There was no macro or microaspiration of microorganisms past the cuff with the PneuX ETT in the stationary, movement or 24 h experiments (Fig. 3).

ANOVA analysis demonstrated a significant difference between all of the studied ETTs when comparing microbial fluid leakage (*p* < 0.00001). The PneuX ETT did not allow aspiration in any experiment and therefore, non-parametric statistical analysis for repeated measures was used to compare all other ETT results against the PneuX ETT. The PneuX ETT demonstrated significantly reduced aspiration compared to the SealGuard (*p* < 0.009), KimVent (*p* < 0.002), TaperGuard (*p* < 0.004), Lanz (*p* < 0.001), ISIS (*p* < 0.001), SACETT (*p* < 0.001) and Soft Seal (*p* < 0.001) ETTs.

### Correlation

**Fig. 4:**
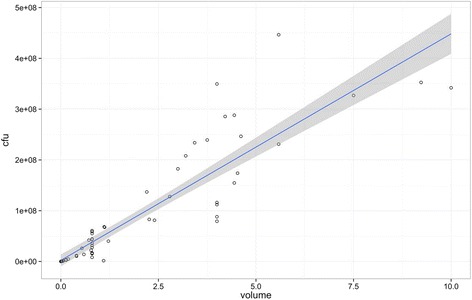
Correlation between volume leaked past the cuff and number of CFU count. Correlation co-efficient = 0.826, *p* < 0.0001

A liner correlation between volume and cfu/min leaking past the cuff was demonstrated, correlation co-efficient 0.826, *p* < 0.0001 (Fig. 4). This high correlation allowed volume of fluid to be used as a surrogate for bacterial load in the effect of ‘tracheal size’ on aspiration experiments.

### Effect of ‘tracheal size’ on aspiration

The 4 ETTs (PneuX, SealGuard, KimVent and TaperGuard ETTs) subjected to the movement test in the microbiological study were selected for testing using different sized ‘trachea models’. Aspiration was expressed as volume of fluid leaked past the cuff.

**Fig. 5:**
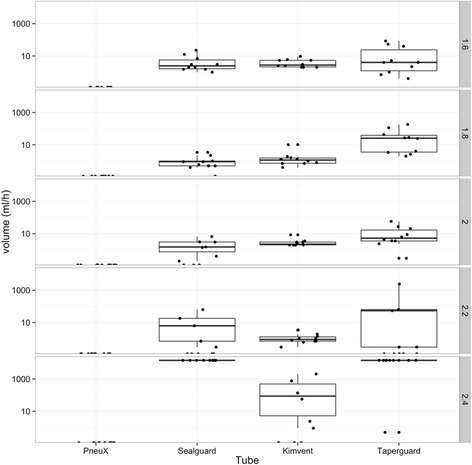
Effect of tracheal size (1.6, 1.8, 2.0, 2.2 and 2.4 cm) on aspiration (volume of fluid) past the cuff per hour. The PneuX ETT was the only tube that prevented the aspiration of fluid at each tracheal size. Variability of the results was evident for each HVLP ETT

With the exception of the PneuX ETT, all ETTs demonstrated leakage of fluid past the cuff across the different trachea sizes. Of the trachea sizes tested, the ‘best’ diameters for the SealGuard and TaperGuard ETTs were the 2.0 and 2.2 cm, and for the KimVent ETT it was 2.4 cm (Fig. 5). Since the PneuX ETT demonstrated no aspiration of fluid past the cuff for any tracheal size non-parametric statistical analysis for repeated measures was again used to compare all other ETT results against the PneuX ETT. The PneuX ETT was found to significantly reduce fluid aspiration compared with the SealGuard (*p* < 0.0001), TaperGuard (*p* < 0.0001) and KimVent (*p* < 0.0001) ETTs.

## Discussion

Aspiration of pathogenic subglottic secretions past the ETT cuff is a recognised risk factor for VAP [9]. All HVLP ETT cuffs form channels allowing aspiration of subglottic secretions [11]. Manufacturers have redesigned aspects of their ETTs to limit this. These studies compared 8 ETTs in preventing aspiration past the cuff in terms of microorganisms and fluid volume. Selected ETTs required a novel design incorporated to prevent subglottic fluid aspiration, with the exception of the Portex Soft Seal, which is one of the commonest ETT in clinical use in the UK.

**Fig. 6:**
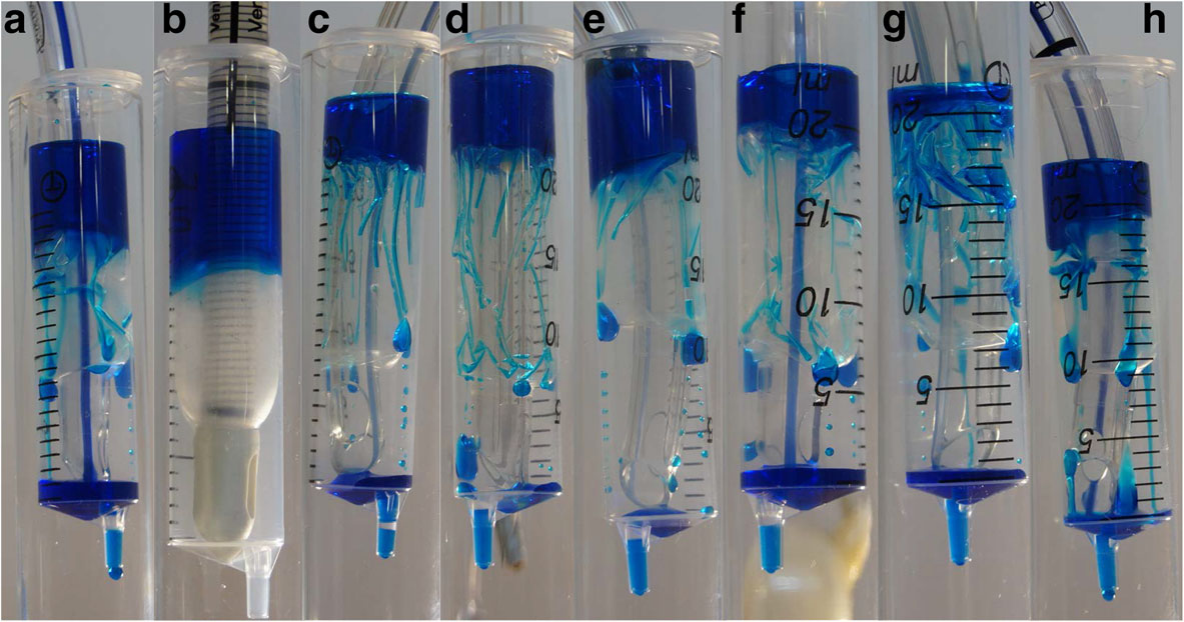
Aspiration of fluid (blue dye) past the cuff and differences in channel formation seen with each ETT cuff. (**a**) Portex Soft Seal ® Cuff Tracheal Tube, (**b**) The Venner™ PneuX P.Y. ™ ETT (**c**) Mallinckrodt™ SealGuard™ Evac Endotracheal Tube, (**d**) KimVent™ MICROCUFF Subglottic Suctioning Endotracheal Tube, (**e**) Mallinckrodt^TM^ Tapergurad™ Evac Oral Endotracheal Tube, (**f**) Mallinckrodt™ Hi-Lo Oral Endotracheal Tube, Mallinckrodt™ Lanz System, (**g**) Teleflex ISIS® HVT™ and (**h**) SACETT™ Suction Above ET Cuff

In this study, all HVLP ETT cuffs exhibited leakage of microbial fluids past the cuff. Only the PneuX ETT (low volume, low pressure (LVLP) cuff), consistently prevented aspiration of fluid under all test conditions (Fig. 3 and 5, 6). Aspiration of microorganisms occurred in all experiments with the Lanz, ISIS, SACETT and Soft Seal ETTs. Results were variable in terms of leaked volume, rate of leakage and microbial aspiration. With the SealGuard, TaperGuard and the KimVent ETTs, some tubes permitted aspiration almost immediately, some progressed to the movement study and some did not leak after manipulation. Similar results were seen for the ‘trachea size’ study, where performance was also variable. The variation within the same type of ETTs, likely relates to the manner in which folds develop within the cuff during inflation, which appears to be random (Fig. 6). This was highlighted in the movement study, where upon manipulation, the folds in the cuffs that had originally formed in such a way to prevent leakage in the static model moved, enabling a fluid pathway to develop thus allowing leakage of fluid. The variability in performance evident in these studies could lead to unpredictable results in clinical practice. A recent clinical study reported that the KimVent, TaperGuard and SealGuard ETTs were not superior to a standard ETT in preventing tracheal colonisation or VAP [17]. Indeed, our results in this in vitro model would have predicted that in a clinical trial [17], where expected duration of ventilation was greater than 48 h the KimVent, TaperGuard and SealGuard ETTs would have not have performed better than a standard ETT in preventing VAP.

Certain HVLP cuff designs are reported to reduce the rate of aspiration and prevent VAP [10, 12–16]. Ultra-thin polyurethane cuffs (wall thickness 7–10 μm) develop finer folds; thereby reduce the rate of aspiration, but not prevent it [13, 32] and this was evident in this study with the KimVent and SealGuard ETTs, which leaked slowest and lowest volumes. Tapered cuffs have reportedly shown benefit over cylindrical cuffs [14, 33]. In this study the TaperGuard and SealGuard ETTs performed better than most HVLP ETTs. However, both the tapered ISIS and SACETT ETT cuffs made of polyvinylchloride, permitted aspiration on all occasions. Polyvinylchloride cuffs (wall thickness 50 μm) [9] develop larger folds which allow greater aspiration of subglottic secretions. This may indicate that cuff material is of greater importance than cuff shape in terms of preventing leakage [32]. The combination, seen in the SealGuard ETT, appears to afford greater protection, as this was the best performing HVLP ETT in this study.

Studies show that maintaining cuff pressure correctly reduces the incidence of VAP [34]. In this study the Lanz and SACETT ETTs were used with their recommended cuff pressure monitors, however their performance was not superior to the Soft Seal ETT. Maintaining correct cuff pressure is important however this study showed that the properties of the ETT cuff may be equally important in preventing aspiration.

The PneuX ETT consistently prevented aspiration in all experiments. The LVLP cuff of the PneuX ETT is made of highly elastic silicone and uniformly inflates until the correct tracheal wall pressure is achieved at the required tracheal circumference, hence, the cuff does not develop folds (Fig. 6) [35]. The PneuX ETT used a tracheal seal monitor, designed to maintain the pressure of the silicone cuff at the appropriate pressure at all times, even during movement [35] and this was demonstrated in this study. Our results replicate previous in vitro studies which demonstrate no leakage past the cuff for the PneuX ETT and superior performance when compared to other ETT examined [10, 36]. Our findings are also supported by a recent publication by Chenelle et al., which demonstrated leakage in a different bench model [37]. In their study, Chenelle et al., showed leakage occurred with all HVLP cuffs tested and was prevented by the PneuX cuff [37]. Our study confirms and builds on this data, by testing all the SSD ETT currently available and also examines the quantification of the bacterial load which the lungs may be exposed to. Commonly, in vitro studies have used water to demonstrate leakage past the cuff [10, 12, 32, 37]. Our study is novel in its use of a microbial solution, which was used to simulate the presence of pathogenic organisms commonly found in the subglottic space. The species used were frequent causative agents of VAP [6, 8]. Clinically microbial counts of 1 × 10^4^ cfu/ml from bronchoalveolar lavages are indicative of VAP [38]. In this study, the microbial solution was equivalent to ca. 10^7^ cells/ml. Although higher than typically encountered clinically, this served to enhance discrimination and sensitivity of the experiments. In experiments where no visible aspiration was seen, the space below the cuff was irrigated to determine whether micro-leaks occurred and in this study, no microaspiration of microorganisms were detected.

In initial experiments, the correlation between cfu/min and volume leaked past the cuff was demonstrated and volume of fluid was therefore used as a surrogate for bacterial load in the ‘trachea size’ study. It was important to test ETTs across a range of trachea sizes, due to the considerable anatomical variability [28]. One may argue that clinicians would not use a size 8.0 ETT in patients with 1.6 or 2.4 cm tracheas. However, patients are not routinely scanned to assess tracheal size prior to intubation and studies have shown the inaccuracies in clinicians assessing tracheal size [30]. Using a size 8.0 ETT in this range of trachea sizes, represents rigorous bench testing and better comparison to clinical practice and anatomical variation.

Many clinical trials with novel ETTs aimed at preventing VAP often show disappointing results. These are costly, time consuming and often fail to replicate in vitro results [39]. Our study tested 8 ETTs across a range of ‘trachea’ sizes, included movement tests and extended durations to increase robustness. ETTs that appear to prevent aspiration in bench tests should be subjected to pilot in-patient studies, where primary outcome measures of aspiration are biomarkers such as pepsin, amylase and bacterial counts [40, 41]. This would demonstrate the adequacy of an ETT to prevent aspiration and would also allow larger clinical trials to be adequately powered.

## Conclusions

Prevention of aspiration past the ETT cuff is a prerequisite for averting VAP. In these investigations, variation in the efficacy of HVLP ETTs was apparent and likely due to the random manner in which folds develop in the cuff. The PneuX ETT was the only ETT to consistently prevent aspiration past the cuff in all experiments. The PneuX ETT should be further evaluated in patients using biomarkers of aspiration to determine whether these results are replicated clinically.

## Abbreviations

AP: Anterior-posterior
ETT: Endotracheal tube
HVLP: High-volume, low-pressure
PBS: Phosphate buffered saline
SSD: Subglottic secretion drainage
VAP: Ventilator associated pneumonia

## References

[1] Melsen WG, Rovers MM, Groenwold RH, Bergmans DC, Camus C, Bauer TT (2013). Attributable mortality of ventilator-associated pneumonia: a meta-analysis of individual patient data from randomised prevention studies. Lancet Infect Dis.

[2] Mietto C, Pinciroli R, Patel N, Berra L (2013). Ventilator associated pneumonia: evolving definitions and preventive strategies. Respir Care.

[3] Young PJ, Doyle AJ (2012). Preventing ventilator-associated pneumonia. The role of the endotracheal tube. Curr Respir Med Rev.

[4] Hamilton A, Grap MJ (2012). The role of the endotracheal tube cuff in microaspiration. Heart Lung.

[5] Coppadoro A, Bittner E, Berra L (2012). Novel preventive strategies for ventilator associated pneumonia. Rev Crit Care.

[6] Chastre J, Fagon JY (2002). Ventilator-associated pneumonia. Am J Respir Crit Care Med.

[7] Metheny NA, Clouse RE, Chang YH, Stewart BJ, Oliver DA, Kollef MH (2006). Tracheobronchial aspiration of gastric contents in critically ill tube-fed patients: Frequency, outcomes, and risk factors. Crit Care Med.

[8] Adair CG, Gorman SP, Feron BM, Byers LM, Jones DS, Goldsmith CE (1999). Implications of endotracheal tube biofilm for ventilator associated pneumonia. Intensive Care Med.

[9] Zolfaghari PS, Wyncoll DL (2011). The tracheal tube: gateway to ventilator-associated pneumonia. Rev Crit Care.

[10] Young PJ, Pakeerathan S, Blunt MC, Subramanaya S (2006). A low-volume, low-pressure tracheal cuff reduces pulmonary aspiration. Crit Care Med.

[11] Deem S, Treggiari MM (2010). New endotracheal tubes designed to prevent ventilator-associated pneumonia: Do they make a difference?. Respir Care.

[12] Lorente L, Lecuona M, Jimenez A, Mora ML, Sierra A (2007). Influence of an endotracheal tube with polyurethane cuff and subglottic secretion drainage on pneumonia. Am J Respir Crit Care Med.

[13] Dave MH, Frotzler A, Spielmann N, Madjdpour C, Weiss M (2010). Effect of tracheal tube cuff shape on fluid leakage across the cuff: an in vitro study. Br J Anaesth.

[14] Shiotsuka J, Lefor AT, Sanui M, Nagata O, Horiguchi A, Sasabuchi Y (2012). A quantitative evaluation of fluid leakage around a polyvinyl chloride tapered endotracheal tube cuff using an in-vitro model. HSR Proc Intensive Care Cardiovasc Anesth.

[15] Carter EL, Duguid A, Ercole A, Matta B, Burnstein RM, Veenith T (2014). Strategies to prevent ventilation-associated pneumonia: the effect of cuff pressure monitoring techniques and tracheal tube type on aspiration of subglottic secretions, an in-vitro study. Eur J Anaesthesiol.

[16] Lucangelo U, Zin WA, Antonaglia V, Petrucci L, Viviani M, Buscema G (2008). Effect of positive expiratory pressure and type of tracheal cuff on the incidence of aspiration in mechanically ventilated patients in an intensive care unit. Crit Care Med.

[17] Philippart F, Gaudry S, Quinquis L, Lau N, Ouanes I, Touati S (2015). Randomized intubation with polyurethane or conical cuffs to prevent pneumonia in ventilated patients. Am J Respir Crit Care Med.

[18] Mahul P, Auboyer C, Jospe R, Ros A, Guerin C, el Khouri Z, Galliez M, Dumont A, Gaudin O (1992). Prevention of nosocomial pneumonia in intubated patients: respective role of mechanical subglottic secretions drainage and stress ulcer prophylaxis. Intensive Care Med.

[19] Kollef MH, Skubas NJ, Sundt TM (1999). A randomized clinical trial of continuous aspiration of subglottic secretions in cardiac surgery patients. Chest.

[20] Lacherade JC, De Jonghe B, Guezennec P, Debbat K, Hayon J, Monsel A, Fangio P, Appere de Vecchi C, Ramaut C, Outin H, Bastuji-Garin S (2010). Intermittent subglottic secretion drainage and ventilator-associated pneumonia: A multicenter trial. Am J Respir Crit Care Med.

[21] Smulders K, van der Hoeven H, Weers-Pothoff I, Vandenbroucke-Grauls C (2002). A randomized clinical trial of intermittent subglottic secretion drainage in patients receiving mechanical ventilation. Chest.

[22] Damas P, Frippiat F, Ancion A, Canivet JL, Lambermont B, Layios N, Massion P, Morimont P, Nys M, Piret S, Lancellotti P, Wiesen P, D'orio V, Samalea N, Ledoux D (2015). Prevention of ventilator-associated pneumonia and ventilator-associated conditions: a randomized controlled trial with subglottic secretion suctioning. Crit Care Med.

[23] Muscedere J, Rewa O, McKechnie K, Jiang X, Laporta D, Heyland DK (2011). Subglottic secretion drainage for the prevention of ventilator-associated pneumonia: a systematic review and meta-analysis. Crit Care Med.

[24] Dezfulian C, Shojania K, Collard HR, Kim HM, Matthay MA, Saint S (2005). Subglottic secretion drainage for preventing ventilator-associated pneumonia: a meta-analysis, Review. Am J Med.

[25] Fernandez JF, Levine SM, Restrepo MI (2012). Technologic advances in endotracheal tubes for prevention of ventilator-associated pneumonia. Chest.

[26] Doyle A, Fletcher A, Carter J, Blunt MC, Young PJ (2011). The incidence of ventilator-associated pneumonia using the PneuX system with or without elective endotracheal tube exchange: a pilot study. BMC Res Notes.

[27] Kollef MH, Afessa B, Anzueto A, Veremakis C, Kerr KM, Margolis BD (2008). Silver-coated endotracheal tubes and incidence of ventilator-associated pneumonia: the NASCENT randomized trial. JAMA.

[28] Kamel KS, Lau G, Stringer M (2009). In vivo and in vitro morphometry of the human trachea. Clin Anat.

[29] Karmakar A, Pate MB, Solowski NL, Postma GN, Weinberger PM (2015). Tracheal size variability is associated with sex: implications for endotracheal tube selection. Ann Otol, Rhinol Laryngol.

[30] D’Anza B, Knight J, Scott-Green J (2015). Does body mass index predict tracheal airway size?. Laryngoscope.

[31] Hollander M, Wolfe DA (1999). Nonparametric statistical methods.

[32] Zanella A, Scaravilli V, Isgro S, Milan M, Cressoni M, Patroniti N (2011). Fluid leakage across tracheal tube cuff, effect of different cuff material, shape, and positive expiratory pressure: a bench-top study. Intensive Care Med.

[33] Lau AC, Lam SM, Yan WW (2014). Benchtop study of leakages across the Portex, TaperGuard, and Microcuff endotracheal tubes under simulated clinical conditions. Hong Kong Med J.

[34] Blot SI, Poelaert J, Kollef M (2014). How to avoid microaspiration? A key element for the prevention of ventilator-associated pneumonia in intubated ICU patients. BMC Infect Dis.

[35] Young PJ (2007). The LoTrach: a tracheal tube for critical care. JICS.

[36] Young PJ, Ridley SA, Downward G (1998). Evaluation of a new design of tracheal tube cuff to prevent leakage of fluid to the lungs. Br J Anaesth.

[37] Chenelle CT, Itagaki T, Fisher DF, Berra L, Kacmarek RM (2017). Performance of the PneuX system: a bench study comparison with 4 other endotracheal tube cuffs. Respir Care.

[38] Morris AC, Kefala K, Wilkinson TS, Moncayo-Nieto OL, Dhaliwal K, Farrell L (2010). Diagnostic importance of pulmonary interleukin-1b and interleukin-8 in ventilator-associated pneumonia. Thorax.

[39] Branson RD, Hess DR (2015). Lost in translation: failure of tracheal tube modifications to impact ventilator-associated pneumonia. Am J Respir Crit Care Med.

[40] Mariyaselvam MZ, Wise MP, Williams DW (2015). Translating in vitro research: improving endotracheal bench test methodology. Am J Respir Crit Care Med.

[41] Jaillette E, Brunin G, Girault C, Zerimech F, Chiche A, Broucqsault-Dedrie C (2015). Impact of tracheal cuff shape on microaspiration of gastric contents in intubated critically ill patients: study protocol for a randomized controlled trial. Trials.

